# Pulmonary Extramedullary Hematopoiesis in a Patient with Chronic Asthma Resembling Lung Cancer: A Case Report

**DOI:** 10.1155/2012/231787

**Published:** 2012-04-03

**Authors:** Massood Hosseinzadeh, Navid Omidifar, Perikala Vijayananda Kumar, Alireza Rasekhi

**Affiliations:** ^1^Department of Pathology, Shiraz University of Medical Sciences, Shiraz 71344, Iran; ^2^Department of Radiology, Shiraz University of Medical Sciences, Shiraz 71344, Iran

## Abstract

*Background*. Extramedullary hematopoiesis is most often seen in reticuloendothelial organs specially spleen, liver, or lymph nodes, and it is rarely seen in lung parenchyma. Almost all reported cases of pulmonary extramedullary hematopoiesis occurred following myeloproliferative disorders specially myelofibrosis. Other less common underlying causes are thalassemia syndromes and other hemoglobinopathies. There was not any reported case of pulmonary extramedullary hematopoiesis in asthmatic patients in the medical literature. *Case*. Here we reported a 65-year-old lady who was a known case of bronchial asthma with recent developed right lower lobe lung mass. Chest X-ray and CT studies showed an infiltrating mass resembling malignancy. Fine needle aspiration cytology of mass revealed pulmonary extramedullary hematopoiesis. The patient followed for 10 months with serial physical examination and laboratory evaluations which were unremarkable. *Conclusion.* Extramedullary hematopoiesis of lung parenchyma can be mistaken for lung cancer radiologically. Although previous reported cases occurred with myelofibrosis or hemoglobinopathies, we are reporting the first case of asthma-associated extramedullary hematopoiesis.

## 1. Introduction

Pulmonary extramedullary hematopoiesis is a rare condition and most commonly occurs in patient, with chronic myeloproliferative disorders specially myelofibrosis with myeloid metaplasia [1]. Other underlying causes include hemoglobinopathies such as thalassemia syndromes, sickle cell anemia, and other chronic hemolytic anemias. We reported an unusual case of pulmonary extramedullary hematopoiesis in an old lady with chronic asthma. The presumptive pathogenetic mechanism for the development of this condition in asthma is the presence of chronic allergen exposure to induce hematopoietic stem cells to the site of inflammation resulting in extramedullary hematopoiesis.

## 2. Case Report

**Figure 1:**
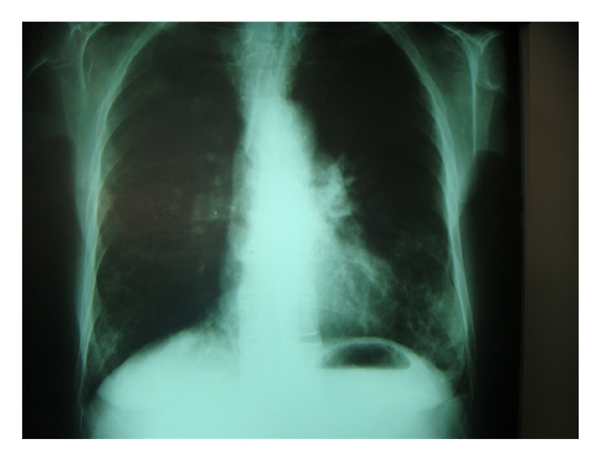
Chest X-ray: infiltration in the base of both lungs.

**Figure 2:**
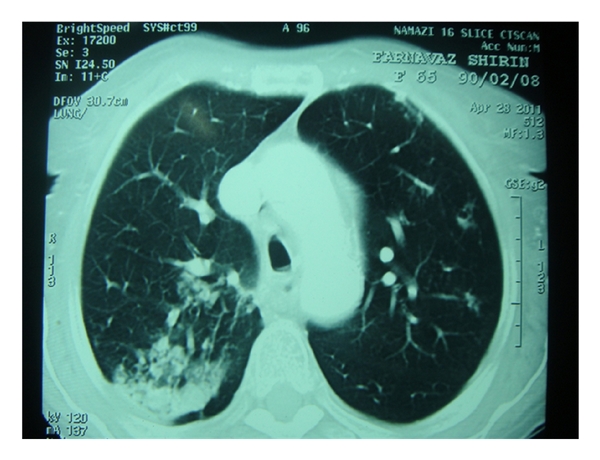
Axial CT scan of the chest, lung window: dense consolidation in the posterior aspect of the right lung. A few other smaller infiltrations are also seen in the left lung in this region. FNA is taken from right side.

**Figure 3:**
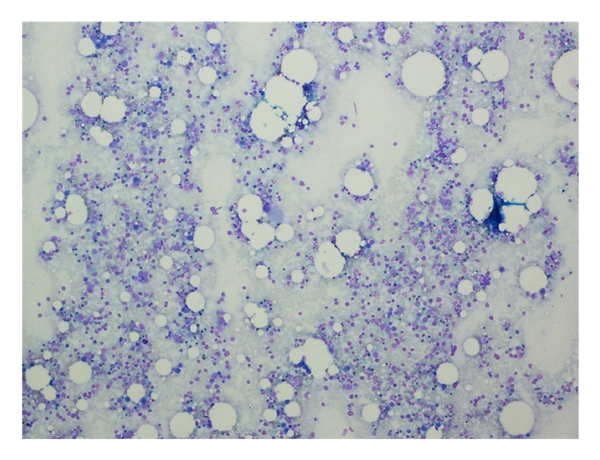
Low-power view of FNA cytology of lung mass shows hematopoetic cells admixed with few fat cells. Wright stain ×100.

**Figure 4:**
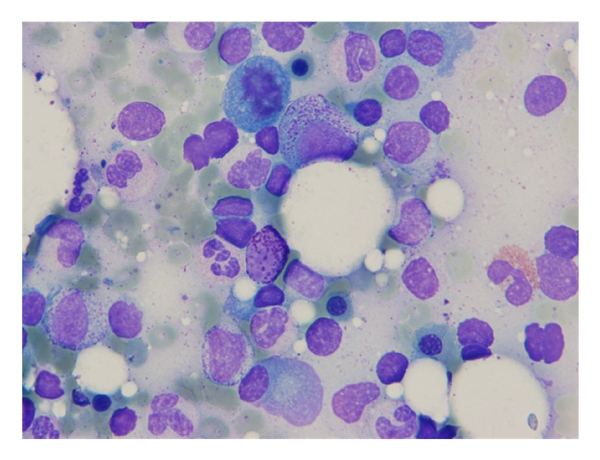
Myeloid and erythroid cells in mid-power microscopic view. Wright stain ×400.

**Figure 5:**
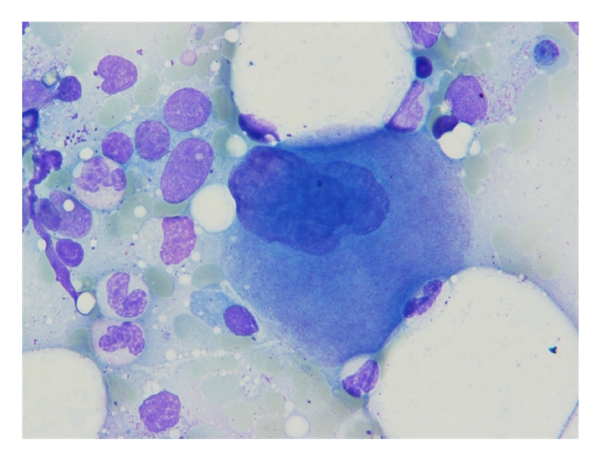
A megakaryocytein final stage of maturation. Wright stain ×400.

A 65-year-old lady was referred to Namazi hospital affiliated to Shiraz University of Medical Sciences because of worsening of her chronic airway disease. She had been confirmed as a typical case of asthma for about 40 years. She was nonsmoker with no history of fever, weight loss, or chest pain. Her past medical history was not significant except for a chronic asthma which was controlled with inhalation of bronchodilator sprays, sodium cromolyn (Intal), and PRN corticosteroids. On physical examination, the general condition was well, with no sign of respiratory distress. The patient was not febrile. There was no lymphadenopathy or hepatosplenomegaly. Chest examination revealed mild wheezing in both lung regions and decreased breathing sounds specially at the right lung base. Laboratory tests showed Hb: 12.8, Hct: 39%, and Leukocyte count: 8900/*μ*L with mild eosinophilia and platelet count: 420000/*μ*L. Serum chemistry profile including liver function tests, FBS, renal function tests, alkaline phosphatase, and calcium was unremarkable. Chest X-ray showed mild hyperinflation and diffuse opacity at the right lower lobe (Figure 1). A contrast-enhanced CT of chest showed an infiltrating mass at the right lower lobe (Figure 2). With the presumptive diagnosis of malignancy fine needle aspiration under guide of CT was performed; the smears were subsequently stained using Wright and modified Papanicolaou methods, respectively. The microscopic examination revealed hypercellular smears composed of islands of erythroid precursors, myeloid series, and some mature and immature megakaryocytes (Figures 3, 4 and 5). Considering the definite diagnosis of pulmonary extramedullary hematopoiesis, bone marrow aspiration and biopsy were performed and showed normocellular marrow without evidence of myelofibrosis in special stains. The patient was followed during a laboratory investigation plan for 10 months with routine treatments for her asthma, but there was not any change toward malignancy or myelofibrosis and any other complications.

## 3. Discussion

Although extramedullary hematopoiesis is a common finding in liver, spleen, or lymph nodes, it is a rare condition reported in the lung parenchyma. Most of the reported pulmonary extramedullary hematopoiesis is seen in myelofibrosis with myeloid metaplasia, a subgroup of chronic myeloproliferative disorders. Rumi et al. presented a case of pulmonary extramedullary hematopoiesis as initial symptom of myelofibrosis. They concluded that early recognition of pulmonary extramedullary hematopoiesis may prevent pulmonary hypertension of myelofibrosis because of early cytoreduction therapy [1]. Kumar et al. also reported a case of pulmonary extramedullary hematopoiesis presented with lung mass which was diagnosed by fine needle aspiration cytology [2]. In another study Hsu et al. explained a case of extramedullary hematopoiesis mimicking metastatic lung carcinoma. The patient presented with a left lower lobe lung carcinoma and left pleural masses, initially thought inoperable metastatic disease radiographically but fine needle aspiration of pleural masses revealed extramedullary hematopoiesis [3]. Pandit et al. and Sehmi et al. in another study showed the significance for mobilization of hematopoietic progenitor cells in allergic inflammation localized in the lung parenchyma of asthmatic patients. The progenitor cells are mobilized from bone marrow and migrate to lung parenchyma [4, 5]. In the current study we reported a case of chronic asthma who was presented with progressive dyspnea and an infiltrating right lower lobe mass diagnosed as extramedullary hematopoiesis by fine needle aspiration cytology. Bone marrow aspiration and biopsy were unremarkable, and 10-month followup of the patient was unchanged. It finally concluded that the possible cause of extramedullary hematopoiesis was most likely due to chronic hypoxia and localized allergic inflammatory cells infiltration and their cytokine secretions which can recruit progenitor cells to that area, causing proliferation of marrow elements. Although asthma is a common disease, such phenomenon is not seen before. We think there are few considerations regarding this rare condition which include chronicity of asthma in this patient and poor disease control possibly due to low socioeconomic condition and being far from good health care. Furthermore, such lesions are more likely seen under microscopic examination, and the tissue biopsy is usually not performed in asthma patients.
